# Evaluation of Integrative Community Therapy with Domestic Violence Survivors in Quito, Ecuador

**DOI:** 10.3390/ijerph20085492

**Published:** 2023-04-12

**Authors:** Chiara Sabina, Diego Perez-Figueroa, Laurent Reyes, Eduardo Campaña Medina, Eluzinete Pereira de Souza, Lisa Markovits, Andrea Carolina Oña Jacho, Gissel Katherine Rojas Bohorquez

**Affiliations:** 1 School of Social Work, Rutgers University, New Brunswick, NJ 08901, USA; 2 Psychology and Behavioral Science Department, London School of Economics and Political Science, London WC2A 2AE, UK; 3 School of Social Welfare, University of California Berkeley, Berkeley, CA 94720, USA; 4 Muyumpa—Community Therapy Training Center, Quito 170516, Ecuador; 5 Pa’Arriba Foundation, Potomac, MD 20854, USA; 6 Centro Ecuatoriano para la Promoción y Acción de la Mujer, Quito 170143, Ecuador

**Keywords:** Ecuador, domestic violence, community support, social support

## Abstract

Integrative community therapy (ICT) is a methodology used in the public health arena to deal with problems facing communities such as depression, substance abuse, and stress. This approach is unique as it builds on critical pedagogy, cultural anthropology, communication, resilience, and systems theory. Additionally, creative arts therapies point to the utility of music as a therapeutic tool. This study employed ICT and a music workshop with domestic violence survivors in Quito, Ecuador, via a pre-post comparison group design. A total of 87 women completed the six-week study—49 in the intervention group and 38 in the comparison group. Measures were taken on self-esteem, general health, resilience, dating violence attitudes, and social support. Additionally, the intervention group answered open-ended questions about their experience, and some participated in a focus group (*n* = 21). The quantitative results indicated that there was improvement in the domains of general health, self-esteem, and social support for the intervention group compared to the comparison group. Themes from the qualitative responses indicated changes in the relationship with the aggressor, psychological and emotional changes, changes in feelings of social support, and changes for the future. The study found promising results for this approach with domestic violence survivors, possibly leading to a community-grounded, non-hierarchical, culturally-responsive intervention for this population.

## 1. Introduction

Violence against women in Ecuador is a prevalent problem that affects 65% of women in their lifetime [1]. Several advances have been made in the country over the last 30 years, including international human rights declarations, national declarations, adoption of a national constitution which prescribes gender equity, and ratification of a criminal code cataloging gender-based violence [2,3,4,5,6,7]. Nonetheless, despite a persistent call for an increase in the quality of services for domestic violence survivors, most services are not sufficiently available, accessible, adaptable, or appropriate [8,9,10]. The most common types of services available include psychological, medical, legal, and social services. While important, these services are critiqued for employing a top-down (from expert to non-expert), colonial, western, medical, and individualistic approach [11]. Here, we evaluate an alternative type of service for domestic violence survivors—integrative community therapy—that overcomes these limitations and is community-based. Our objective is to evaluate this approach on the outcome variables of self-esteem, general health, resilience, dating violence attitudes, and social support using a pre-post comparison group design, supplemented by focus groups.

### 1.1. Description of Integrative Community Therapy

Dr. Adalberto Barreto created integrative community therapy (ICT) in 1986 in Brazil to address community violence and related problems in favelas [12]. Unlike other interventions, ICT is an inexpensive form of community intervention run by community members with basic training (The ICT training is about 240 h that covers ICT theory, practice exercises, personal healing, and facilitation of 30 dialogue circles. Training is approved by ABRATECOM [Brazilian Association of Community Therapy]). In contrast to models which situate knowledge and expertise in experts, ICT sees knowledge as arising “from the base, in the base, for the base” [13]. Barreto critiqued the medical model, intent on discovering the biological links between symptoms and illness, as being focused on the disruptive effects of illness rather than the illness itself; he argued that a biopsychosocial model [14] would be more adequate in understanding the individual in context. ICT seeks to foster autonomy on an individual and systemic level by understanding the problem in community. It expands upon the patient/professional relationship of the medical model by fostering personal growth, development of relationships, social inclusion, and accountability through listening to common pains. ICT incorporates several theoretical pillars, including cultural anthropology, critical pedagogy, communication theory, systemic thought, and resilience.

With regard to cultural anthropology, ICT explicitly embraces culture (defined as a cluster of artistic practices, knowledge, and tools), which is often discounted in medical, colonial, and imperialist models. Valuing and empowering marginalized, minoritized, and oppressed communities can relieve suffering brought on by cultural imposition [15]. Cultural identity is central to ICT, as culture is considered healing [16,17], and the approach uses songs, sayings, poems, local spiritual and medicinal traditions, local celebrations, and other cultural tools to help rediscover and strengthen cultural identity. ICT seeks to honor traditional and ancestorial forms of knowledge as a way of reconnecting individuals to faith in themselves and their cultures [18]. Postcolonial and decolonial voices argue for *grupalidad curadora*, loosely translated as “group healing” [11], which contributes to a personal and community reconfiguration that exists apart from a medical model and requires a more holistic, transdisciplinary approach.

Many aspects of Paulo Freire’s critical pedagogy and methodology have been adapted to the ICT framework. For Freire, the teaching process is always political, and could either uphold or change the social structure. Pedagogy is an exercise in dialogue, exchange, and reciprocity. Accordingly, there is a time to talk and a time to listen, a time to learn, and a time to teach [19]. It is emancipatory to understand communication and teaching as a horizontal rather than a vertical process [20]. This generates free dialogue, capable of joining theory and practice, producing knowledge and consciousness that promotes a critical understanding in the individual [21]. By knowing what we are, why we are that way, and appropriating our own experience, we become masters of our life and not victims of others or circumstance, thus creating a more just and freeing existence.

Barreto included Paul Watzlawick’s views on communication theory in the ICT methodology [22] and considered good communication a means to a better life. Furthermore, he included Watzlawick’s five axioms as basic rules for communication [23] when participating in ICT. Generally, all actions, even when people chose not to act, are considered communication and point to some important message that should be understood (Rule 1). Communication serves the purpose of providing information about the self and responses to the communication can either confirm or negate the speaker’s self-image (Rule 2). Effective communication requires a mutual understanding between both parties (Rule 3), a combination of both verbal and non-verbal communication (Rule 4), and can be symmetrical or complementary (Rule 5). There are certain conversational rules in ICT, such as listening when others are speaking, only speaking about one’s own personal experience, and avoiding counseling or judgement [18].

General systems theory [24] proposes an orientation toward individual problems within a broader context that takes into account interpersonal relationships, community relationships, and social systems. Likewise, it focuses on the interconnections of the parts of a system in order to understand human suffering, instead of a linear model [25]. Thus, ICT asks participants to look at problems within their context in order to address them.

An objective of ICT is to foster resilience [12], which is understood as the ability to come through a highly disruptive situation and be able to return to relatively stable, healthy levels of psychological and physical functioning [26]. According to Barreto [12] resilience must be built on multiple forms of knowledge and cultural expressions, which can make ICT more effective than other forms of therapy, given that not all trauma experiences can be accessible through verbal processes [27]. As Tummala-Narra [28] said, “Expressions of resilience are influenced by one’s identifications with a cultural group, relationships with family, and interactions with mainstream culture”. Resources such as supportive relationships and strength such as interpersonal and meaning-making strengths improve paths toward trauma recovery and resilience [29]. Through ICT, it is possible to create such an environment.

Consequently, in ICT, these theories consolidate into a method of interpersonal and inter-community encounters. Participants use their life stories to reaffirm their identities by perceiving problems and possible solutions through local resources. It is a therapeutic act moving away from a model focused on pathology and toward a promotion of health and social inclusion. This process is led by a community therapist whose role is to be aware of the objectives and limits of ICT. The therapist does not solve problems but rather creates an environment in which participants can share experiences and build a support network. The therapist does not impose their own solutions or thoughts but instead generates ideas within the group [12].

ICT incorporates six stages within each session: reception, choice of theme, contextualization, problematization, closure, and appreciation/evaluation [25]. The first stage, reception, involves facilitating a sense of belonging, camaraderie, and friendship among all group members. This gives way to the second stage, choice of theme, which activates participants’ agency through the opportunity to propose and select topics of discussion that are relevant to their present situations and concerns. The third stage, contextualization, is designed to clarify the situation/problem within the emotional, relational, and historical context, as well as discuss the ways the participants have overcome it. The fourth stage, problematization, honors participants’ lived experiences and resilience, and identifies individual and group resources that help participants increase their capacity to resolve identified problems or difficult situations. Through this, the therapist formulates a question so that the participants can participate in collective thought and reflection. This is not pushing the group to give advice but instead to share experiences and support as a community. Subsequently, the therapist closes the session (fifth stage) by reframing the topic that was presented, acknowledging the participants’ effort and bravery, and recognizing the desire to overcome difficulties. This positive reframing in the sixth stage enables the participants to rethink and reconsider their suffering in a broader sense.

### 1.2. Evidence of ICT Effectiveness

Is there evidence that ICT is an effective treatment? The extant research is mostly centered on Brazilian samples, spanning a variety of topics: mental health and empowerment among women [30], substance dependence [31], and psychological care centers [32]. In public health, ICT has been studied as a means to deal with stress [33] and strengthen social bonds [34] and as an all-around tool used for psychosocial intervention in a community [35]. ICT has been used with expectant mothers [36], older adults [37], and college students [25]. While ICT has not been systematically applied to women suffering gender-based violence, domestic violence and other forms of violence often surface as topics [12,25]. A meta-analysis of ICT research centered on health promotion included 15 studies from 2008 to 2018 and concluded that it is an effective methodology across different populations, having found a positive impact on emotional/mental health in 58% of the studies [38]. Another recent review by Guimarães Lemes et al. [39], which included 17 studies, found ICT to be a helpful methodology across a range of mental health issues. It must be noted, however, that the methodologies used in the studies did not provide for rigorous evaluations and instead focused largely on interviews and ICT meeting summary sheets, thus offering limited empirical testing. In both reviews all studies were published in Brazil, mostly within nursing, and relied on qualitative methodologies. One nursing study, however, employed a pre-post test design (no comparison group) and found that ICT was associated with a reduction of depression over a five-week period for girls [40]. Overall, more rigorous testing of ICT is needed.

Given the theoretical foundations of ICT, along with the promising evidence found for various populations, we sought to evaluate this approach with domestic violence survivors. We supplemented the ICT approach with a music workshop, as creative arts therapies have been found to be useful for coping with trauma [41]. Here, we used music as a way to provoke reflection on gender roles and serve as a launchpad for discussion, analysis, and reflection as advocated in the creative arts tradition [41]. Several factors lead to the assumption that these approaches would be beneficial for this population including the utility of group approaches with this population, a general lack of formal help-seeking, and the common discussion of violence in dialogue circles [25,42,43]. Using a pre-post comparison group design, we assessed baseline and subsequent general health, self-esteem, resilience, acceptance of dating violence, and social support. With this approach, we would expect to see a more marked improvement in the intervention group as compared to the comparison group over the six-week period, if in fact the method was effective with domestic violence survivors.

## 2. Methods

This study was conducted using a pre-post comparison group design in Quito, Ecuador, in the summer of 2021. In partnership with several local community organizations, such as CEPAM (Ecuadorian Center for the Promotion and Action of Women), Warmi, Muyumpa—Community Therapy Training Center, and Fundación Pa’Arriba Ecuador, we developed and implemented an intervention for survivors of domestic violence based on a modified music workshop that employed ICT (one session) and ICT circles (five sessions). The modified music workshop included a thematic ICT session in which participants listened to an original song on gender roles and then held a discussion on creating new gender roles. All other sessions followed the traditional ICT method and topics of conversation were not directed.

CEPAM is a well-recognized organization in Ecuador dedicated to achieving gender equality and preventing gender violence and serves more than 70 women per month in Sangolquí and Quito. Warmi seeks to serve the population of Pichincha on issues of gender violence against LGBTIQ persons, women, and children and adolescents. Muyumpa is an ICT training center in Quito, Ecuador, operating since 2014, and is part of the Brazilian Association of Community Therapy. Fundación Pa’Arriba is a nonprofit organization that identifies and implements innovative community interventions to reduce domestic violence.

### 2.1. Participants

Sociodemographic characteristics of participants in the study are shown in Table 1. In general, participants self-identified as ethnically mestizo (93%), aged between 19 to 39 years (84.4%) and single, divorced, or separated (69.7%). The level of education in this sample is considered high; 49.9% indicated having completed university or technical school or had started or finished postgraduate studies. Most of the participants indicated at the beginning of the intervention that they had worked the previous week (39.5%) or that they were students (24.4%). Approximately 35% of the participants reported having been in two violent relationships and 23.34% reported three or more violent relationships. Among the most recent relationships that included violence, 52.3% indicated that it had been with a stable partner, 27% with a cohabiting partner, and 19% with a spouse. The most common type of violence reported was psychological/emotional; all participants in the sample reported having experienced this type of violence. The second most commonly experienced type of violence was physical abuse (73.6%). Most of the participants reported that violent relationships had lasted more than three years (58.6%). About 24% reported that they had ended the violent relationship at least one year before the start of the study.

### 2.2. Measures

#### 2.2.1. Health

To measure participants’ health, the study used the General Health Questionnaire (GHQ) [44], which is designed to screen for psycho-emotional problems. The 12-item scale asks about mood and concentration in the last week. The participants were instructed to respond by selecting one of four responses for each question: *much less than usual*, *the same than usual*, *more than usual*, *or much more than usual*. This scale has been previously translated to Spanish and found to have adequate reliability and robust validity among a Spanish population [45]. In our sample, we found the scale to have a Cronbach’s alpha of 0.92 using SPSS 28.

#### 2.2.2. Self-Esteem

The Rosenberg scale [46], which has been previously translated into Spanish and validated with women in violent relationships [47,48], was used to measure self-esteem. This study used a short form of the questionnaire that included 10 statements about a sense of confidence and self-worth [47]. For each statement, the participants could select one of four responses: *strongly disagree*, *disagree*, *agree*, *or strongly agree*. This gave the scale a range of 10 to 40, with higher scores indicating greater levels of self-esteem. For our sample, this scale had a Cronbach’s alpha of 0.85 using SPSS 28.

#### 2.2.3. Social Support

The Social Provisions Scale (SPS) was used to measure changes in level of social support [49,50]. This scale includes 24 items measuring interpersonal support, with higher scores indicating more support. However, for this study, a shorter version of the scale translated to Spanish was used [51]. The questionnaire includes 10 statements about the perception of support available. For each statement, participants could select *strongly disagree*, *disagree*, *agree*, *or strongly agree*. In our sample, the Cronbach’s alpha for this measure was 0.86 using SPSS 28.

#### 2.2.4. Resilience

Resilience was measured using the Brief Resilience Scale (BRS), which is designed to assess an individual’s ability to recover from a stressful or traumatic experience [52]. The BRS questionnaire includes six statements about the ability to recover from a difficult situation. Respondents indicated the degree to which they agreed with each statement on a 5-point scale ranging from *strongly disagree* to *strongly agree*. This scale has been previously translated into Spanish and found to have adequate reliability and validity among a Spanish speaking population [53]. Our alpha was 0.80 using SPSS 28.

#### 2.2.5. Dating Violence Attitudes

The questionnaire measuring acceptance of dating violence (ADV) focused specifically on types of behaviors in a relationship. This questionnaire includes nine items assessing participants’ attitudes about acceptable or unacceptable behaviors in a relationship and the questionnaire has been validated in Spanish [54]. Participants could rate their agreement of each item using a 6-point scale that ranged from *completely false* to *completely describes me*. This scale was found to have adequate reliability and validity. Our sample was found to have a Cronbach’s alpha of 0.80 using SPSS 28.

#### 2.2.6. Participant Feedback

The post-survey included open-ended questions for participants to elaborate on their experiences and give suggestions to improve the intervention in the future. Six qualitative questions were asked: 1. Has the relationship with the aggressor changed during the time of this study? If the answer is yes, how has the relationship with the aggressor changed? 2. How has participating in the music workshop and the dialogue circles helped you today? 3. How could this experience benefit you in the future? 4. Which experience from the music workshop and the dialogue circles do you remember the most? 5. What would you change about the music workshop and the dialogue circles? 6. Would you recommend this experience to other women who have experienced intimate partner violence? In addition to these questions, a focus group was conducted with 21 participants who received the intervention. The same questions were used to guide the discussion.

### 2.3. Procedure

Recruitment of participants was facilitated through announcements from CEPAM, Warmi, and Facebook. CEPAM contacted potential participants by phone and selected them for the study. The screening interview included questions about their demographics, Internet access, past experiences of domestic violence, current violent experiences, and access to a safe and private environment to facilitate participation during the six-week online program. We recruited women who had experienced violence but were no longer in the violent relationship. To account for attrition, we recruited 130 women to participate in the study (75 in the intervention group and 55 in the comparison group). Participants elected if they would enroll in the intervention or comparison group according to their interest and availability. Once capacity was reached for the intervention group, we only offered placement in the comparison group. A total of 87 completed the study and both pre- and post-tests—with 49 in the intervention group and 38 in the comparison group.

The intervention consisted of six meetings that were held over Zoom once a week for 1.5 h during July and August of 2021 following the session format of ICT. For these meetings, the intervention group was broken down into four smaller groups (18 to 26 people) to accommodate varying schedules and to allow for group cohesion. Groups were run by certified ICT therapists with extensive experience with the approach. Additionally, in June 2021 we had an introductory meeting for each group in which participants completed the preliminary survey, and we had another meeting at the end of August in which participants completed the post-survey. The comparison group met twice, once for pre-tests and once for post-tests, six weeks apart, with no intervening contact. Participants in the comparison group received a USD 20 incentive for their participation; those in the intervention group received a USD 10 incentive for every week they attended, amounting to a total of USD 60 for completing the program. Incentives were provided via bank transfers. A meta-analysis has found incentives to be helpful in recruitment and retention [55] and other studies with immigrant Latinas in the USA have also compensated participants [56].

All procedures performed in this study were approved by the Institutional Review Board of the University of Delaware on 17 November 2020 (Study Number 1449966). In addition, the study implemented several strategies to ensure the safety of participants. First, the study screened for women who were previously but not currently in abusive relationships, had a private and safe space to speak freely, and were not in crisis. For those enrolled in the study, additional help was available through CEPAM, Warmi, and other community resources. The comparison group was offered CEPAM and Warmi services at the beginning (but not ICT) and ICT circles after the study completed. We also encouraged all participants to continue with services they were already receiving or to seek out additional services as needed.

### 2.4. Data Analysis

An analysis comparing the intervention group and the comparison group was carried out via *t*-tests (for continuous variables) and chi-square tests (for categorical variables) to capture any statistically significant difference between the groups. Quantitative data analysis was carried out on SPSS 28 and relied on a two-way (intervention/comparison) repeated measures (pre/post) ANOVA to ascertain the effectiveness of ICT. Although there were significant differences between groups at baseline, control variables were not entered into the analysis, given power issues. The qualitative analysis consisted of coding themes [57] that emerged throughout participants’ answers to the six open-ended questions in the post-survey (changes with perpetrator, changes in self, changes for future). Codes were assigned and reviewed by another researcher until 100% agreement was reached. In addition, the participants evaluated the program, highlighting what they liked the most and what needed improvement. The results of the focus group (*n* = 21) expand on each topic, complementing the results of the open-ended questions. Coding of open-ended responses and focus group data was completed in Spanish and translated for the purposes of presentation here.

## 3. Results

### 3.1. Comparison between Groups

There were no significant differences in characteristics of age, ethnicity, education, employment, or duration of the violent relationship between the experimental and comparison groups. However, significant differences were noted between the groups in the domains of relationship type, number of violent relationships, types of violence experienced, and services accessed. Results from an independent *t*-test showed that the intervention group had more violent relationships (*M* = 2.52, *SD* = 1.89) than the comparison group (*M* = 1.68, *SD* = 0.99), *t* (74) = 2.64, *p* = 0.01. Significant differences were noted in two types of violence between the groups. The intervention group had significantly higher percentages of sexual abuse (63%) than the control group (42%), *χ*^2^ (1, *n* = 87) = 3.858, *p* = 0.05 as well as a higher percentage of reproductive abuse (22%) compared to the control group (5%), *χ*^2^ (1, *n* = 87) = 4.974, *p* = 0.01. Results from a chi-square test showed differences in types of relationship between the control and intervention group, *χ*^2^ (5, *n* = 86) = 13.683 *p* = 0.02, as well as differences in types of services sought, *χ*^2^ (7, *n* = 87) = 15.17 *p* = 0.03. Overall, the comparison group was more likely to be in a relationship during the intervention compared to the intervention group, who were more likely to be divorced. Finally, the results indicated significant differences in types of services attended between the groups. The intervention group reported more frequent use of therapeutic support groups (88%) compared to the control group (11%). Finally, the comparison group reported more frequent use of the police (13.2%) compared to the intervention group (4%). No significant differences were noted in the type of partner in the most recent violent relationship or the length of time they were in that relationship.

### 3.2. Quantitative Results

A series of two-way repeated measures ANOVAs were used to examine if participants in the intervention and comparison groups differed on their changes on each of the outcome variables over time. Regarding main effects for time, resilience (*F* (1) = 8.84, *p* = 0.004), health (*F* (1) = 19.97, *p* < 0.001), social support (*F* (1) = 24.77, *p* < 0.001), and self-esteem (*F* (1) = 16.68, *p* < 0.001) scores varied across the two time points. There was a statistically significant difference in health (*F* (1, 1) = 19.04, *p* < 0.001), social support (*F* (1, 1) = 8.10, *p* < 0.01), and self-esteem (*F* (1, 1) = 3.98, *p* < 0.05) across time between the intervention and comparison groups. There were no statistically significant changes evident for dating violence attitudes and resilience. See Table 2 for descriptive statistics and condition (intervention, comparison) * time (pre-, post-) results.

**Figure 1:**
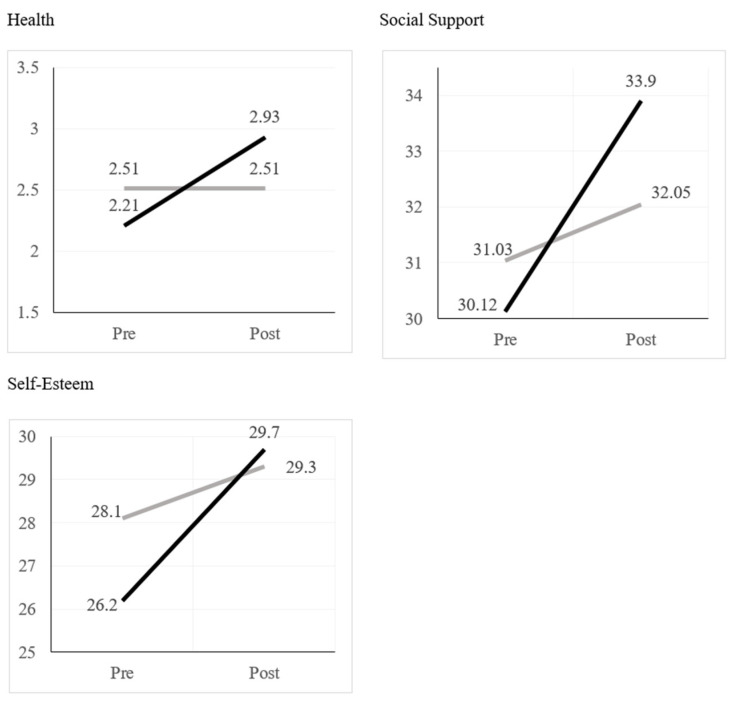
Significant Interactions Between Group and Time for Health, Social Support, and Self-Esteem. Note: Solid black lines represent the intervention group and the gray lines represent the comparison group.

Significant interaction effects are shown in Figure 1. For health, there was significant improvement across time for the intervention group (*F* (1, 46) = 32.94, *p* < 0.001) but not for the comparison group (*F* (1, 37) = 0.01, *p* = 0.93). Partial eta squared for this interaction was 0.19, representing a large effect size. For social support, there was significant improvement across time for the intervention group (*F* (1, 47) = 27.74, *p* < 0.001) but not for the comparison group (*F* (1, 36) = 3.09, *p* = 0.09). Partial eta squared for this interaction was 0.09, representing a medium effect size. For self-esteem, there was significant improvement across time for the intervention group (*F* (1, 48) = 17.46, *p* < 0.001) but not for the comparison group (*F* (1, 37) = 2.67, *p* = 0.11). Partial eta squared for this interaction was 0.05, representing a small to medium effect size. Thus, across health, social support, and self-esteem there were significant improvements for the intervention group, while the comparison group did not significantly change.

### 3.3. Qualitative Results

#### 3.3.1. Theme 1: Changes in the Violent Relationship

Most of the participants had already distanced themselves from their aggressor; approximately 69% reported being single, divorced, or separated. Therefore, the majority of participants declared that the program had not changed the violent relationship because they had already established limits and distance with the aggressor. As an example, one participant wrote:

*“I do not have a current relationship with my aggressor, neither verbal and much less physical. Since we finished, I have focused on my space, my well-being and getting out of all of that, completely removing him from my life and starting to live from scratch.”*

The rest of the answers highlighted that the intervention had helped to establish limits with the aggressor and put distance (*n* = 10) and another three indicated that the relationship had not changed since they were still involved due to custody or social circle issues. Of the participants who responded that the intervention had helped to change the relationship with the aggressor, one described how her participation had helped her to set limits on her current relationship. She wrote:

*“I have imposed limits, I recognize his victimhood so that he sometimes recognizes that he needs help, I feel more sure of myself, with the strength that if there are no changes, if I can get out of there, I no longer feel alone.”*

More common were responses that mentioned that the intervention helped them completely cut off communication with the aggressor. For example, one participant wrote, “*I used to reply to your messages, since you always wanted me to forgive you. I have forgiven him, but I prefer not to answer anything.*” Other participants said, “*I no longer have contact with him*” and “*I have lost interest in his life*”. These results show that the intervention has the potential to change the violent relationship in a positive way, such as establishing limits and distance. One of the participants expanded on this topic during the focus group, highlighting the music methods used in the intervention:

*“Until now, I always take a song from Lu that said “I sway, but I don’t fall. I sway, but I don’t fall.” So every time I have a difficulty, I remember that and I know that I can sway, but I’m not going to fall. And well, this circle of help also came at a very difficult time for me, very difficult, with my aggressor. We were closing an important issue and I was too scared to talk to him. It scared me a lot. But this helped me understand, because in each circle we talk a little about what I had already experienced, remember that there are difficult things and I will overcome them. I knew how to handle it and I’m still here, and that also helped my self-esteem knowing that I can make my decisions, for myself, without fear of anyone.”*

#### 3.3.2. Theme 2: Psychological and Emotional Changes

The psychological and emotional changes that resulted from the intervention were the most notable. More than 70 answers across all the questions talked about changes in this area. For example, approximately 37 responses highlighted how the intervention helped participants increase their self-confidence, self-love, and hope that they could overcome violent relationships. A repeated phrase was “*keep going*”, which denoted the feeling of empowerment, strength, and security that many felt at the end of the study. For example, one participant spoke of how the intervention had helped her find the support she needed to “*move on*”. She wrote: “*First, it helped me a lot to seek help, to fill myself with courage and get ahead and realize that I am a woman who has a lot of worth and is capable of moving forward despite everything.*”

Participants also wrote about the changes they had noticed in their feelings of guilt, shame, and resentment toward the aggressor. Approximately 33 responses included issues of healing and clarity about the violent relationship. For example, many commented on a change in awareness about what they had experienced and how this change helped them in the process of emotional healing. One participant wrote, “*Now I am aware that I still have the wound from that relationship and that I must acknowledge that wound and continue to heal it.*” Likewise, another participant wrote that her awareness of the shame she felt had changed thanks to the intervention: “*I understood that I should not be ashamed of my situations, there are many people who experience something similar, and I could see options to resolve things.*”

Many other participants spoke about the release of guilt they felt by participating in the intervention and talking with others who had been through similar situations. As one participant wrote: “*It helped me realize that I am not the only person who goes through this and to see that I can overcome everything, not being alone is important and lose the shame of being judged.*”

Increased self-esteem and self-confidence resulting from the intervention were also highlighted. For example, one participant wrote:

*“It has helped me a lot to understand self-love, the many ways in which I can get ahead and that this reality is more common than normal but that I am not alone and that I can trust people. I can help and I can help myself, so that this never happens to me again and I can finally be happy with my new life loving myself.”*

One participant expanded on the topic of self-love as an integral aspect of the healing process to recover her emotional and psychological well-being during the focus group. She said:

*“I believe that this workshop that we have followed and of which I have been a part has helped me a lot to embark on a path of many things. I have realized mistakes that I have made and that, more than anything, I have lacked self-love. Realize that I have to learn to forgive myself. If I’ve made mistakes, then that’s life, right? In other words, the important thing is to move forward and improve as human beings. So, I take all that with me and it has served me a lot, it has motivated me a lot to see life differently, with more optimism, being more positive. That has helped me.”*

These results present further evidence for the results that emerged from the quantitative analysis which demonstrate significant increases in the areas of self-esteem and general health. In the words of one of the participants, “*I felt happy, I had fun, I discovered healing and joy. I have felt personal acceptance, I lost the fear of being judged. It improved my self-esteem and mental health.*”

#### 3.3.3. Theme 3: Changes in Feelings of Social Support

As many of the responses above point out, social support was an essential element of the intervention that helped the participants feel heard and accompanied in their experiences of violence. A phrase that was repeated in many responses was “*I am not alone*”, which reinforces how important it was to have a group of people who had gone through situations similar to them, in which they had felt very alone and isolated. For example, one participant wrote:

*“They have helped me a lot, many times I blamed myself too much and thought I was the only one who had suffered. After listening to them I feel stronger, empowered and more self-esteem (which I didn’t have much of before).”*

Like her, many others shared the feeling of learning from other women and feeling heard, and that this was the first time they had been able to speak freely about their experiences. For example, a participant commented:

*“I felt that I was no longer alone, I was able to talk about topics that I did not talk about with anyone, they listened to me and supported me, I also felt peace and singing the songs or doing exercises outside during my daily life prolonged the effect of peace and company.”*

Since the intervention used music as a method to process the trauma of the violent relationship, many participants spoke of how it helped their emotional state: “*The circles and the music workshop have allowed me to share experiences, let me know that I am not alone, I have proven that talking heals.*”

The realization that they are not alone helped participants to increase their self-confidence and give them strength to overcome the situation: “*It has allowed me to understand other experiences of women who have lived through the same situation. On many occasions it gave me strength.*” Likewise, a participant in the focus group spoke of the importance of a support group with whom she can vent and talk about the experiences of violence she has suffered:

*“I think I learned a lot because my fear was always speaking and telling my experience and feeling judged. Because that is what happened to me at the beginning when I spoke and told what had happened to me. People usually always said to me, “But why didn’t you speak up?” But when I had the opportunity to talk about it here, no one said it that way, instead they understood it. They understand the experiences we live, each one of us is healing at their own pace. I know that I lack many things to let go of, but it is the first time that I spoke about it in a group with other women where I actually felt loved from a distance. It was also very nice to experience this circle of support and listening, which is something that I had not experienced and had not had within reach….”*

#### 3.3.4. Theme 4: Changes for the Future

Although the music and ICT workshops only lasted six weeks, the results show that the effects have potential beyond the duration of the program. Participants expressed in their qualitative responses that they felt less ashamed of their situation and more confident in their ability to avoid becoming involved in violent relationships in the future. Approximately 18 responses addressed this topic; one sentiment that came up often was “*Don’t fall into another toxic relationship*”. Thus, one achievement of the program was to help the participants feel able to avoid violent relationships in the future. For example, one participant wrote, “*It definitely helps me relate in any area of my life, and not let myself fall into violent relationships. Never doubting my self-esteem has risen and I feel very good.*” Similarly, another participant highlighted several areas where she felt the program helped her: “*To be more sure of myself in how I project myself with the rest. To not depend emotionally on my future partner. To be more empathic with people. To not be afraid, nor repress who I am.*”

A desire to help other people in violent relationships also emerged, since participants had experienced the benefit of having social support in these situations. Several participants shared the feeling that “*we can help more women in similar situations*” as one said. In general, they expressed a sense of community that they can draw upon in the future. For example, one participant said, “*It can benefit me by reminding myself that I am not alone and that talking is healing. My testimony can serve others.*” In addition, another participant talked about how she can continue to foster empathy and responsiveness skills with other women who are in violent relationships. She said, “*To continue building empathy with other women and affected people, experiencing violence. To know how to contain and accompany victims of this violence without re-victimizing them.*”

Thus, the program has the potential to build community and support beyond the people directly involved. This theme also emerged during the focus group. One participant commented on the need to continue programs that create a space for learning and healing, where women who have suffered violence can take an active role in their own healing process as well as help other women going through the same thing:

*“I really liked the experience, and I have also talked with my friends who have been through the same thing. It is necessary to have these spaces to feel safe, with empathy. There are many times that we don’t talk to other people because they can’t understand us because they haven’t been through the same thing. I feel that this is very important in these groups. We know how we feel. We empathize with each other because we’ve been through it. Only we can understand everything we’ve been through.”*

## 4. Discussion

The results of this pilot study show a statistically significant change in the lives of women who have suffered domestic violence in Quito, Ecuador, through a six-week music and ICT intervention program. The quantitative results indicate improvement in the domains of general health, self-esteem, and social support. The largest effect size was for health, particularly for a sense of meaning and worth, confidence, and ability to enjoy life. The other two quantitative changes on social support and self-esteem mirror this trend. These results were reinforced by the qualitative comments offered by the participants in their open responses and during the focus group. Similarly, qualitative responses demonstrate changes in attitudes toward domestic violence and improvements in resilience, although the quantitative analysis did not capture these changes.

This study represents a more rigorous evaluation of the ICT methodology; previous studies employed largely qualitative methodologies [38,39]. As such, this study appears to be unique in its quasi-experimental multi-method approach. Our implementation was also unique in that we started sessions with an original song and a thematic ICT circle, which allowed participants an opening to begin to express themselves in a non-threatening and engaging way. We then followed this by five traditional ICT circles in which themes were decided collectively. The evaluation shows ICT to be supportive and helpful in similar ways as indicated by previous evaluative work. Themes of self-worth, social support, and healing were common throughout the quantitative and qualitative results.

Certain aspects discussed by the participants mirror the theoretical orientation of the ICT approach. For example, a shared appreciation of songs reinforced the cultural groundings of the methodology, which seeks to enrich cultural appreciation. The results also reflect the approach’s focus on promoting autonomy, building self-esteem, and collective group efficacy. Participants pointed to their location within systems—relationships to aggressors and others who have suffered violence. The communal nature of the program is of utmost importance as it moves from self-healing to group healing and encourages participants to be active rather than passive subjects [25]. This is best reflected in the qualitative responses, in which participants repeatedly pointed to their ability to confront and deal with the problems resulting from experiencing domestic violence. However, in these admissions, the participants always underscored the importance of group solidarity to help them to overcome their difficulties. Speaking to others who had similar backgrounds helped survivors find their own voice. Group formats using a trauma-informed cognitive behavioral program have been found to be equally effective for IPV survivors compared to individual formats [47]. Other researchers have found that domestic violence support groups allow survivors to establish trust and build social networks [58]. Thus, there is additional evidence pointing to the utility of the group approach for domestic violence survivors.

## 5. Conclusions

ICT has largely been absent from discussions of partner violence and testing it adds new possibilities. The method closest to this approach in the published literature is the community health worker/promotora model, in which a member of the target community delivers health education in clinical and non-clinical settings to peers [59]. This model has been tested with immigrant Latina survivors in the US and was found to contribute to self-empowerment [60]. There is, however, a difference in the objective and delivery of information. Promotoras share their knowledge of public health issues with participants. Here, facilitators accompany participants on issues of importance to them. ICT also differs from other group therapy approaches in that with ICT, there is no clinician and no necessity of a presenting issue. A non-professional, who is trained in ICT, facilitates the group and relies on a horizontal rather than vertical format. It is the collective experience and sharing within and among peers that creates the momentum for self-confidence and change. This aspect, along with others, that speaks to the social justice and liberatory dimensions of ICT and separates it from other group formats and promotora models.

One of the main ways in which ICT embodies social justice and liberation, unlike the medical model, is by starting with the assumption that solutions lie in the community. There is no outside imposition of a solution: local knowledge, as opposed to academic knowledge, is valued [18]. Another aspect is its accessibility—there is no insurance, money, diagnoses, or, in many cases, transportation needed as ICT occurs in community settings [18] and is led by community therapists. The approach thus can reach a large number of people in a direct way. The adaptability of the ICT methodology to domestic violence survivors speaks to its versatility to address a variety of social problems [25,39].

There are considerable limitations to this study. First, our intervention and comparison groups statistically differed from the beginning of the study, but our sample sizes were not large enough to include control variables in analyses. Given the interest in our study, the intervention group filled up before the comparison group. We did not randomly assign people to the experimental or comparison group and instead relied on their availability, along with the capacity of the project to accommodate survivors in the intervention group. Another limitation was the resilience measure, as it relied on a personal characteristic of being able to bounce back quickly from adversity. This conception of resilience runs counter to current work focused on a range of strengths and resilience promoting factors and to the experience of domestic violence survivorship, which can entail a long arduous journey. In line with this process, it may be necessary to run this intervention for longer time periods for stronger effects and/or to continue assessment of outcome variables over time, perhaps on a monthly basis. Given that some of the quantitative analyses were underpowered, we suggest that future work replicate this evaluation with larger sample sizes and perhaps with additional comparisons to other methodologies. While methodologically random assignment is best for detecting differences between groups, we felt that that approach was not appropriate for DV survivors, especially when the comparison group did not receive any intervention. Comparisons of different approaches may be better suited for random assignment.

From this study, it appears ICT is a promising methodology that could extend beyond the reach of formal services for domestic violence survivors. A majority of domestic violence survivors do not seek formal services, leading those in the field to argue for a network-oriented approach to domestic violence practice [61]. This approach, which could include peer support groups, substantially broadens the possibilities of engagement for domestic violence survivors. Thus, ICT could be implemented in connection with traditional domestic violence services, as well as through non-violence-related community agencies, or through personal networks. The flexibility and adaptability of the approach are useful for a variety of settings, populations, and locations and it warrants further study.

## Figures and Tables

**Table 1:** Demographic Characteristics (*n* = 87).

	*n*	%
Age (*n* = 64)		
19–29	33	51.6%
30–39	21	32.8%
40+	10	15.6%
Ethnic Identity (*n* = 86)		
Indigenous or indigenous nationality	1	1.2%
Montubia	2	2.3%
Mestiza	80	93%
White	3	3.5%
Civil Status *(n* = 86)		
Married	3	3.5%
Civil union	2	2.3%
In a relationship	21	24.4%
Single	34	39.5%
Separated	13	15.1%
Divorced	13	15.1%
Level of education (*n* = 86)		
Completed primary school	1	1.2%
Completed secondary school	16	18.6%
Did not complete university or technical school	18	20.9%
Completed university or technical school	34	39.5%
Did not complete postgraduate degree	3	3.4%
Postgraduate degree completed	6	7.0%
Other (including currently in school)	8	9.3%
Employment in the previous week (*n* = 86)		
I was a student	21	24.4%
I worked	34	39.5%
I didn’t work but I have a job	2	2.3%
I was looking for work for the first time	1	1.2%
I was looking for work that I did previously	12	14.0%
I did domestic labor	7	8.1%
I have a physical or mental limitation that does not allow me to work	3	3.5%
I did not work	6	7.0%
Number of violent relationships (*n* = 86)		
1	36	41.9%
2	30	34.9%
3 to 5	17	19.8%
More than 5	3	3.5%
The most recent relationship that included violence (*n* = 86)		
Casual dating partner	2	2.3%
Steady partner	45	52.3%
Partner I was living with	23	26.7%
Husband	16	18.6%
Type of violence in the last relationship (*n* = 87)		
Physical	64	73.6%
Sexual	47	54%
Reproductive	13	14.9%
Psychological or emotional	87	100%
Economic	43	49.4%
Stalking	46	52.9%
How long was your last relationship that included violence *(n* = 87)		
Less than a year	13	14.9%
1–2 years	23	26.4%
3–5 years	26	29.8%
6–9 years	14	16%
10+ years	11	12.6%
When did your last violent relationship end (*n* = 87)		
Currently in the relationship	1	1.1%
Less than six months ago	12	13.8%
6 to 11 months ago	8	9.2%
1–2 years ago	30	34.5%
3–5 years ago	20	22.9%
6+ years ago	16	18.3%
Services used to help with partner violence (*n* = 82)		
Social work	4	4.9%
Psychologist or mental health professional	51	62.2%
Attorney or public defender	2	2.4%
Group therapy	9	11%
Judicial system	8	9.8%
Civil rights protection	1	1.2%
Police	7	8.5%

**Table 2:** Descriptive Statistics by Condition and ANOVA Results.

		Pre	Post	*F*	*p*
Dating Violence Attitudes	Intervention	1.11	1.01	3.04	0.09
	Comparison	1.21	1.25		
Resilience	Intervention	16.08	18.04	0.25	0.62
	Comparison	16.21	17.61		
Health	Intervention	2.21	2.94	19.04	<0.001
	Comparison	2.50	2.51		
Social Support	Intervention	30.13	33.90	8.10	0.01
	Comparison	31.03	32.05		
Self-esteem	Intervention	26.20	29.65	3.98	0.05
	Comparison	28.08	29.26		

## Data Availability

The data are not publicly available due to the sensitive nature of the data collected. Please contact the corresponding author should data be requested.
